# Dementia and Its Implications for Public Health

**Published:** 2006-03-15

**Authors:** Daniel P Chapman, Sheree Marshall Williams, Tara W Strine, Robert F Anda, Margaret J Moore

**Affiliations:** Division of Adult and Community Health, Centers for Disease Control and Prevention; Division of Adult and Community Health, Centers for Disease Control and Prevention, Atlanta, Ga; Division of Adult and Community Health, Centers for Disease Control and Prevention, Atlanta, Ga; Division of Adult and Community Health, Centers for Disease Control and Prevention, Atlanta, Ga; Division of Adult and Community Health, Centers for Disease Control and Prevention, Atlanta, Ga

## Abstract

**Introduction:**

With the aging of the U.S. population, a better understanding of the presentation and impact of dementia is essential to the future of public health. Dementia refers not to a single disorder but to a number of syndromes characterized by diverse behavioral, cognitive, and emotional impairments. Because dementia is costly in terms of both personal suffering and economic loss, an understanding of its prevalence, risk factors, and potential interventions is emerging as an increasingly important facet of public health and health care delivery. Recent advances in the understanding of its presentation, course, and relevant interventions have taken place.

**Methods:**

We identified articles for review primarily by conducting a Medline search using the subject headings *dementia, mild cognitive impairment, Alzheimer's disease, vascular dementia, frontotemporal dementia,* and *Lewy body dementia*. Other relevant studies were elicited through a Medline search using the subject headings *mental disorders* and *stigma.*

**Results:**

Dementia represents a diverse category of syndromes characterized by deficits in memory, cognitive function, and behavior. Symptoms associated with dementia appear to be distributed along a continuum, with even subsyndromal presentations affecting the health of older adults and meriting intervention. To promote cognitive functioning and independence among older adults, public health interventions need to facilitate both early detection and treatment of dementia. The availability of adult day care and respite services is important in maintaining the health and quality of life of individuals caring for older adults with dementia. Recent advances in the treatment of dementia may slow the course of cognitive decline, thereby enhancing the quality of life of older individuals as well as decreasing costs associated with institutional care.

**Conclusion:**

Despite the growing availability of pharmacologic and psychosocial interventions that are potentially helpful to people with dementia and their caregivers, the majority of older adults with dementia do not receive appropriate treatment. With the aging of the U.S. population, efforts to foster recognition of dementia and its treatments and to destigmatize them are emerging as an increasingly important facet of public health intervention.

## Introduction

Projected demographic changes in the U.S. population suggest that a better understanding of psychiatric disorders among older adults is of vital importance to public health. As summarized elsewhere (1), the combination of a declining birth rate and an increased average life span is expected to increase the proportion of the population aged 65 years and older in the United States from 12.4% in 2000 to 19.6% in 2030. The number of people aged 65 years and older is expected to increase from 35 million in 2000 to 71 million in 2030. The number of people aged 80 years and older is also expected to double, from 9.3 million in 2000 to 19.5 million in 2030. Globally, the number of older adults (aged 65 years and older) is projected to increase even more dramatically — more than doubling from 420 million in 2000 to 973 million in 2030 (1).

The publication of *Mental Health: A Report of the Surgeon General* indicates that psychiatric disorders and their prevention are important facets of public health. In the chapter devoted to older adults and mental health, the report emphasizes that older adults can benefit from recent advances in the treatment of psychiatric disorders and that these advances can prevent disability and promote the autonomy of older adults (2). Similarly, the report of the President's New Freedom Commission on Mental Health, *Achieving the Promise: Transforming Mental Health Care in America,* recommends that mental health be addressed with the same urgency as physical health (3).

It has been reported that in any given year, nearly 20% of older community dwellers have a psychiatric disorder (2), with estimates increasing to approximately 90% of older nursing home residents (4). This article provides an overview of one of the most common psychiatric disorders among older adults, dementia, and examines its presentation, prevalence, treatment, and public health implications.

## Methods

Articles included in this review were primarily identified through a Medline search of the terms *dementia, mild cognitive impairment, Alzheimer's disease, vascular dementia, frontotemporal dementia, Lewy body dementia, mental disorders,* and *stigma*. Studies retained for review were generally limited to empirical investigations that provided definitional or diagnostic criteria for the disorders and that specified an interval of observation.

## Results

### Dementia

Dementia is a syndrome characterized by cognitive or memory impairments not involving any alteration in consciousness or alertness. The cognitive impairment characterizing dementia may include memory loss, difficulty in understanding or using words, inability to carry out motor activities despite adequate motor function, and failure to identify or recognize objects (5). People with dementia commonly experience impairments in occupational and social functioning (6) and may present behavioral disturbances (7). 

The prevalence of dementia has been estimated to be approximately 6% to 10% of individuals aged 65 years or older; prevalence increases with age, rising from 1% to 2% among those aged 65 to 74 to 30% or more of those aged 85 or older (8). In another investigation, 40% of those aged 90 to 94 were reported to suffer from dementia, with the prevalence of dementia peaking at 58% among individuals older than 94 years (9). It is noteworthy that, at present, the majority of older adults do not experience dementia. Nevertheless, with older adults projected to represent a greater proportion of the U.S. population, the cost of caring for people with dementia will become an increasingly important public health consideration. Dementia increases the mean annual health care cost per older patient by $4134, with 75% of these increased costs attributable to increased hospitalization and expenditures on skilled nursing facilities (10). Not all forms of dementia are irreversible, however, and new interventions may forestall further decline in functioning among people with dementia. Timely recognition and intervention are key to the optimal care of older adults with dementia, which may be attributable to a number of causes.

**Reversible causes of dementia**

A complete medical history, physical examination, and medication review are important components of the initial assessment of individuals presenting the memory and cognitive impairments characterizing dementia. Vitamin deficiencies, thyroid dysfunction, and normal-pressure hydrocephalus (the accumulation of cerebrospinal fluid [CSF] that enlarges spaces within the brain) have all been identified as potentially reversible causes of dementia (11). Although reversible causes of dementia comprise only about 9% of cases of dementia and appear to have decreased in prevalence (12), identifying and treating potentially reversible causes of dementia remains of utmost importance (13).

**Depressive symptoms**

Depressive disorders may feature cognitive deficits similar to those observed in dementia. However, in delayed recognition tests, individuals with dementia were reported to be more likely to make intrusion errors (false positive errors), while individuals with depression tended to underreport recognition of items actually presented (false negative errors) (14). In a related investigation, depression was associated with early morning awakening and fragmented sleep, whereas dementia was associated with deficits in rapid eye movements during sleep and with sleep-disordered breathing (15). Increased anxiety and decreased sex drive were found to be more common among depressed individuals, whereas those with dementia were found to manifest greater disorientation to time and difficulty completing self-care tasks (16). Individuals whose cognitive decline is attributable solely to depression may experience restoration of cognitive function once their depression has been effectively treated (17).

The relationship between depression and dementia is complex and not entirely understood. Early depressive symptoms among older adults with mild cognitive impairment have been found to be a risk factor for subsequent development of dementia (18). Notably, older adults with depressive symptoms and cognitive impairment have been reported to have abnormalities in brain structure (19) and cerebral blood flow (20) similar to abnormalities observed in older adults with dementia. Treatment of depression may, however, prevent further functional deterioration among people with the "depression-executive dysfunction" syndrome of late life (21).

**Delirium**

Dementia must also be distinguished from delirium, a condition characterized by impairments in consciousness and attention, as well as by the impairments in memory and cognition observed in dementia. The onset of delirium is usually abrupt, whereas the presentation of dementia is characteristically insidious (5). Delirium can be precipitated by illness or intoxication, or it can be induced by medication (22). Often presenting as an acute confusional state (22), delirium is a medical emergency (23) associated with a high risk for morbidity and mortality (22). Unlike dementia, however, delirium typically resolves if the underlying cause is effectively treated (24), although older adults may be slow to return to their premorbid level of functioning (23). In addition to receiving treatment for its underlying cause, individuals with delirium may gain symptomatic relief if they are provided with a comfortable environment and, if needed, antipsychotic medication (24). These findings underscore the necessity of an initial medical evaluation of older adults experiencing cognitive impairment.

### Mild cognitive impairment

Efforts to better understand memory or cognitive impairments among older adults not meeting criteria for dementia have expanded markedly in the last decade (25). Older adults experiencing mild cognitive impairment (MCI) typically present with subjective memory complaints (ideally verified by an informant) and also show objective evidence of memory deficits as measured by neuropsychological tests. However, the degree of memory or cognitive impairment experienced by people with MCI does not interfere with their ability to perform activities of daily living (26). In a multicenter population study of older adults, 19% of participants younger than 75 and 29% of participants older than 85 were identified as having MCI (27). Longitudinal research has revealed that between 23% and 47% of adults aged 75 and older who were initially dementia free but who manifested "ageing associated cognitive decline" (a construct similar to MCI except that it does not require subjective memory impairment) developed dementia over a 2.6-year period (28).

Individuals with MCI who have the epsilon 4 allele of *APOE*, the gene for apolipoprotein E, appear to be at increased risk of progressing to dementia (29). The Quality Standards Subcommittee of the American Academy of Neurology recommends that individuals with MCI receive clinical monitoring and evaluation, including neuropsychological testing (26). Although MCI has been proposed as a potential starting point for intervention (30), the effectiveness of treatments for MCI is currently under evaluation (26).

### Alzheimer's disease 

Researchers have estimated that approximately 75% of individuals with dementia have Alzheimer's disease (AD) (31). AD can be distinguished from other forms of dementia by its insidious and progressive course, although plateaus in the progression of AD may occur. Depression, insomnia, incontinence, delusions, and hallucinations may be manifest as the disease progresses, as may neurologic signs including sudden muscle contraction and gait disturbance (32). Because its initial presentation is subtle, information provided by someone close to the individual with mild AD may be vital to its early recognition (33). Impairments in recall of items on the Mini-Mental State Examination and names of relatives, difficulty with calculation (such as balancing a checkbook or maintaining household finances), making repetitious statements, getting lost while driving, and exhibiting poor judgment are among the constellation of symptoms associated with early AD (34).

Characteristic brain pathology of AD includes the presence of neurofibrillary tangles (interweavings of filaments within the body of the nerve cell [neuron]) and plaques indicative of neuron degeneration (35). Degeneration is particularly prominent among neurons that release the neurotransmitter acetylcholine (36). Abnormalities in the transport of glutamate, the chief excitatory neurotransmitter in the brain, may also underlie the development of AD (37).

Recent research has suggested that the CSF of patients with AD has altered levels of proteins associated with neuron degeneration. However, the utility of CSF as a risk indicator for AD awaits further evaluation (38). To date, identified risk factors for AD include a positive family history of the disorder (39-42), limited education (39,42,43), head injury (39,42), *APOE* genetic endowment (41,43), and age (40,42,43).

### Vascular dementia 

Vascular dementia (VaD) has been estimated to account for 15% to 20% of all dementias among older adults (44) and is precipitated by some form of cerebrovascular disease (45). Most commonly, blockage of blood vessels in the brain yields the death of tissue, or *infarction*, in the affected region. Infarction underlying dementia may involve a single, strategic blood vessel or numerous smaller ones (multiple infarct dementia). Traditionally, VaD has been characterized by sudden onset (44), stepwise progression (44,46), and focal neurological deficits (44,46) associated with the region of the brain affected. However, results of research undertaken during the last decade have revealed that an estimated 20% of cases of VaD are characterized by an insidious onset and a steadily progressive course (47). Postmortem examinations of the brains of individuals with dementia have suggested that the coexistence of VaD with AD is not uncommon (48).

Laboratory tests and brain imaging techniques can be used in the diagnosis of VaD (47). Hypertension (49-51), diabetes (52), age (53), atherosclerosis (54), and male sex are probable risk factors for vascular dementia (55). Because several of these factors have also been associated with an increased risk of AD (54,56), recognition of potential vascular components of AD is growing (57).

### Frontotemporal dementia 

Primarily affecting the regions of the brain governing planning, social behavior, and language perception, frontotemporal dementia (FTD) includes the syndrome commonly referred to as *Pick's disease *(58). Relative to the other dementias, FTD is characterized by a younger age of onset, with its presentation after age 75 being rare (59). FTD can also be distinguished from other dementias by a distinctive constellation of symptoms. In contrast to AD, older adults with FTD initially manifest less memory impairment (60) but more changes in speech and personality (60), indicative of disinhibition and poor social awareness (61).

The behavioral presentation of FTD may include inappropriate swearing, impulsive decisions and purchases, repetitive actions, and inappropriate sexual behavior. Changes in eating habits and deficits in self-care may also be present (59). FTD may also include progressive deterioration of language function. Older adults with FTD may present with difficulties in word usage, precipitating reading and writing impairments that may culminate in mutism (59). Recent research has suggested FTD may be more prevalent than was previously believed (62,63). A family history of dementia and mutation of the gene that produces tau protein have been associated with FTD (64).

### Lewy body dementia 

As its name implies, Lewy body dementia (LBD) is characterized by the presence of Lewy bodies, proteins in the cerebral cortex (which governs thought processing) and brain stem (which coordinates movement). Lewy bodies are also common among individuals with Parkinson's disease. Although prevalence estimates vary, some researchers have estimated that LBD accounts for 15% to 20% of all cases of dementia (65,66). As might be expected, people with LBD have been found to manifest signs of parkinsonism (67), such as difficulties in initiating movements, slowness of movement, muscular rigidity, and tremor.

Although its clinical presentation may be similar to that of AD, individuals with LBD can be distinguished from individuals with AD by marked cognitive fluctuations, prominent hallucinations (68), and the presence of parkinsonism (67). Recent research suggests that LBD can also be differentiated from AD by behaviors that the individual exhibits during periods of cognitive slowing. Daytime drowsiness despite adequate sleep, sleeping for 2 or more hours during the day, staring into space for long intervals of time, and periods of nonsensical or disorganized speech are more common in LBD than AD (69).

In addition to the presence of Lewy bodies, pathology characteristic of AD (i.e., plaques, and, to a lesser degree, neurofibrillary tangles) is also frequently present in LBD (70). However, distinguishing these two forms of dementia is crucial, because individuals with LBD are highly sensitive to the adverse effects of antipsychotic drugs (65,70), which may be administered with the intent of providing relief from distressing hallucinations.

### Dementia interventions 

Although dementia remains a source of great suffering for many older adults, the results of recent research suggest that there are reasons for optimism about the prospects of improved care of older adults with cognitive impairment. Some forms of dementia, albeit a minority, can be reversed through timely intervention. Moreover, not all forms of cognitive impairment among older adults are, in fact, dementia. Some are attributable to other conditions that are amenable to treatment. Public health efforts to heighten awareness of the importance of early evaluation of older adults showing signs of cognitive impairment are clearly warranted, as are efforts to educate the public about the heterogeneity and potential reversibility of cognitive impairment. Perhaps most significantly, dementia is increasingly recognized as the endpoint of a continuum of cognitive decline among older adults, with the detection of mild cognitive impairment suggesting new opportunities for intervention.

**Medications**

The destruction of neurons releasing the neurotransmitter acetylcholine appears to be common among people with AD and some other dementias. By blocking the enzyme responsible for breaking down acetylcholine, medications designed to inhibit cholinesterase have been found to increase acetylcholine levels in the brain (71). The efficacy of these drugs appears to be greatest during the early phases of dementia, before the receptors of the neurons they target have been destroyed (72). Notably, cholinesterase inhibitor use has been reported to delay initial nursing home placement by approximately 21 months among individuals with AD (73) as well as to reduce behavioral symptoms of dementia (74).

Similarly, memantine, a drug approved by the U.S. Food and Drug Administration (FDA) for moderate to severe AD, has been shown to regulate receptor activity affecting the neurotransmitter glutamate, which is also implicated in AD (75). Among individuals already receiving a cholinesterase inhibitor, those who were also given memantine were found to have better outcomes than those receiving only a cholinesterase inhibitor (76).

**Vitamins and nonsteroidal anti-inflammatory drugs**

Researchers have also examined the effect of antioxidants — compounds believed to combat a variety of degenerative diseases — on the development and progression of dementia. Studies of vitamins E and C have yielded mixed results. Some investigators found that neither supplemental nor dietary intake of vitamins E or C reduces users' risk of VaD or AD over an interval of several years (77,78). However, the results of a related investigation suggest that dietary vitamin E is associated with a reduced risk for AD but only among people without the *APOE* epsilon 4 allele (79), while other research indicates that dietary intake of vitamins C and E is associated with reduced risk for AD (80). In addition, vitamin E and C supplements have been reported to reduce the risk for VaD but not AD among Japanese American men (81), and higher doses of these vitamins have been associated with a decrease in the number of incident cases of AD (82). Results of recent research suggest that taking vitamin E and C supplements together may reduce the users' risk for AD (83) and that vitamin E may slow the progression of AD (84,85), although its utility in delaying or preventing AD among individuals with MCI has not been fully evaluated (85). The variability of study results suggests that the ability of antioxidants to reduce the risk for dementia or to delay its onset may depend on an interaction of individual and biochemical characteristics not yet fully elucidated.

Because of evidence that an inflammatory process may underlie the development of AD (86,87), the potential use of nonsteroidal anti-inflammatory drugs (NSAIDs) in the prevention and treatment of dementia has also been examined. Results of community-based investigations have shown that the prevalence of AD among individuals reporting NSAID use was lower than the prevalence observed among individuals who did not use NSAIDs (88,89). In other studies, the protective effect of NSAIDs against AD was not found to be dose related or to extend to VaD (90). As the duration of NSAID use increases, the risk for developing AD is reduced (91,92); long-term use of NSAIDs was found to reduce users' risk for AD, but only if NSAID use was initiated before the onset of dementia (93).

Some research suggests that NSAIDs may ameliorate some aspects of cognitive impairment. Although the results of one placebo-controlled trial did not show that administration of an NSAID significantly reduced the rate of cognitive deterioration among individuals with AD (94), the results of other studies showed a slower rate of decline among NSAID users in specific aspects of cognition, such as associative learning (95), verbal fluency, spatial recognition, and orientation (96). However, NSAID use has also been associated with adverse effects, including fatigue, hypertension, dizziness (97), and abdominal pain (94), resulting in significant withdrawal rates. Selective cyclooxygenase-2 (COX-2) inhibitors have not been demonstrated to slow the cognitive decline characteristic of AD (97).

**Mental and physical activity**

Approximately one third of decedents found to have moderate AD neurofibrillary pathology through postmortem examination of the brain  did not manifest memory impairment during their lifetime. It has been proposed that this apparent resistance to dementia may reflect the presence of *cognitive reserve* (98). This theory is consistent with the results of previous research that found that increased education exerted an apparent protective effect, decreasing the risk for AD (39,42,43).

Reading, playing games or puzzles (99-101), and playing a musical instrument (101) have been associated with a decreased risk for subsequent dementia among older adults free from cognitive impairment. In general, physical activity has not been associated with a similarly decreased risk for dementia (100,101), although the results of one study found that higher levels of physical activity relative to no exercise were associated with a lower risk of cognitive impairment, AD, or any dementia (102). Moreover, it has been contended that physical activity in older adults increases blood flow to the brain, thereby helping to preserve cognitive function (103); in one study featuring older adults with cardiac disease who manifested varying degrees of dementia, walking was found to improve cognitive performance (104).

Because motor performance impairment (105) and changes in activity rhythm (106) may be distinguishing signs of dementia among older adults, physical activity has been investigated as a means of improving the physical and mental status among people with dementia. Physical activity among individuals with AD has been associated with increased appendicular skeletal muscle mass, which promotes functional autonomy (107). Similarly, regular physical exercise, such as walking or using an exercise bicycle, has been shown to improve the nutritional and cognitive status and decrease the risk for falls and behavioral problems among people with AD (108). Physical activity, in conjunction with behavioral techniques to optimize functioning, has also been associated with improvements in physical health and a reduction in depressive symptoms among people with AD (109).

### Caregivers of people with dementia

Unquestionably, caregivers assume a vital role in the well-being of people with dementia by helping them with appropriate nutrition and exercise, providing them with memory aids, and assisting with behavioral interventions (110). Several studies have attempted to identify characteristics associated with caregivers' risk of developing depressive symptoms. Health (111,112), caregiving competence (112), and attitude toward asking for help (111) have been associated with depressive symptoms among caregivers.

Dependency on the caregiver to perform instrumental activities of daily living (113), depression (113-115), and problematic behaviors (114,116) are characteristics of people with dementia that are also associated with increased depressive symptoms or added burden among caregivers. This association appears to be particularly noteworthy, as caregiver depression commonly precipitates the discontinuation of home care of disabled older adults (117).

Teaching caregivers strategies for managing the behavioral problems of individuals with dementia (118), and, in some cases, providing case management and community services for individuals with dementia (119) have been reported to decrease depressive symptoms among caregivers. Similarly, providing adult day services for individuals with dementia has been shown to reduce the time caregivers spend on problematic behaviors (120). The provision of respite care for individuals with dementia has been shown to decrease caregivers' stress (121) and enhance their quality of life (122).

### Public health interventions 

The Alzheimer's Association and the American Association of Retired Persons (AARP) have both launched initiatives designed to foster public awareness of strategies to preserve and enhance cognitive function among older adults. Both of these initiatives seek to promote cognitive functioning rather than ameliorate serious cognitive deficits. The Alzheimer's Association's initiative, *Maintain Your Brain*, encourages older adults to "make brain-healthy life choices," such as staying physically, mentally, and socially active and consuming a low-fat diet rich in antioxidant-laden fruits and vegetables (123). Similarly, the AARP's initiative, *Staying Sharp*, encourages participants to engage in "learning throughout life" and teaches them techniques useful in "minding your memory," such as repetition, visualization, building associations, and planning and prioritizing (124). *Staying Sharp* also offers 2-hour forums in selected cities to discuss these issues. Although neither of these interventions appears to have yet been formally evaluated, their promise is suggested by the success of cognitive training in improving the cognitive performance of older, independent-living adults (125).

Although preventing or forestalling the cognitive and behavioral deficits characterizing dementia remains an important goal for public health, the need for services for people with dementia and their families will likely remain very strong. The U.S. Administration on Aging assists state and area agencies on aging in the delivery of support services such as respite and day care both to optimize the care received by people with dementia and to ease the burden placed on their caregivers (126). The Alzheimer's Association offers related assistance, such as support groups for caregivers and locator devices to identify the whereabouts of individuals with dementia who may be missing (127).

### Stigma 

Although interventions can frequently improve the functioning and quality of life of people with dementia, the dementia of many older adults remains undiagnosed and untreated. Both health care providers and the general public may stigmatize older adults with psychiatric disorders (128), and negative attitudes toward the mentally ill have been associated with a lack of knowledge about mental illness among older adults (129). The stigma associated with psychiatric disorders has been shown to have serious consequences for the health of people with mental illness, including decreased self-esteem (130) and a lower sense of psychological well-being and life satisfaction (131). A survey of mental health care consumers revealed that the majority attempted to conceal their disorders and feared that disclosure of their psychiatric status would precipitate unfavorable treatment by others (132).

## Discussion

Dementia is associated with significant disability and impaired quality of life among older adults. Dementia encompasses an array of syndromes featuring some distinct forms of presentation posing important implications for intervention. Public health efforts designed to foster awareness of the signs of cognitive impairment among older adults are needed, as early intervention may forestall further decline in cognitive functioning. Essentially, older adults, their health care providers, and others around them need to be better informed that dementia is not an *expected* aspect of aging, but rather a *real *disorder amenable to intervention. Recent research results suggest that pharmacologic and psychosocial interventions may forestall cognitive decline among people with dementia, provided they are implemented early in the course of the disease. Caregivers of individuals with dementia are at increased risk for significant depressive symptomatology, although adult day care and respite care appear to be important resources to help them reduce that risk. Related efforts are needed to destigmatize dementia and its treatment, thereby removing significant barriers to the continued health and functioning of older adults.
